# Molecular and Cellular Response to Experimental *Anisakis pegreffii* (Nematoda, Anisakidae) Third-Stage Larval Infection in Rats

**DOI:** 10.3389/fimmu.2018.02055

**Published:** 2018-09-07

**Authors:** Ivana Bušelić, Željka Trumbić, Jerko Hrabar, Anamarija Vrbatović, Ivana Bočina, Ivona Mladineo

**Affiliations:** ^1^Institute of Oceanography and Fisheries, Split, Croatia; ^2^Department of Marine Studies, University of Split, Split, Croatia; ^3^Faculty of Science, University of Split, Split, Croatia

**Keywords:** RNA-Seq, transcriptome, accidental host, anisakiasis, immune response, proinflammatory

## Abstract

**Background:** Anisakiasis is a zoonotic disease caused by accidental ingestion of live *Anisakis* spp. third-stage larvae present in raw or undercooked seafood. Symptoms of this emerging infectious disease include mild-to-severe abdominal pain, nausea, and diarrhea. Some patients experience significant allergic reactions.

**Aims:** In order to better understand the onset of anisakiasis, we aimed to: (i) histopathologically describe severe inflammatory/hemorrhagic infection site lesions in Sprague-Dawley rats experimentally infected with *Anisakis pegreffii* larvae; and (ii) qualitatively and quantitatively characterize the transcriptomes of affected tissues using RNA-Seq.

**Methodology:** The experiment was performed on 35 male rats, sacrificed at 5 time points (6, 10, 18, 24, and 32 h post-infection). Gastric intubation was performed with 10 *A. pegreffii* larvae (*N* = 5 infected rats per time point) or 1.5 ml of saline (external control *N* = 2 rats). 16 pools, seven for muscle tissues and nine for stomach tissues, were created to obtain robust samples for estimation of gene expression changes depicting common signatures of affected versus unaffected tissues. Illumina NextSeq 500 was used for paired-end sequencing, while edgeR was used for count data and differential expression analyses.

**Results:** In total, there were 1372 (855 up and 517 down) differentially expressed (DE) genes in the *Anisakis*-infected rat stomach tissues, and 1633 (1230 up and 403 down) DE genes in the muscle tissues. Elicited strong local proinflammatory reaction seems to favor the activation of the interleukin 17 signaling pathway and the development of the T helper 17-type response. The number of DE ribosomal genes in the *Anisakis*-infected stomach tissue suggests that *A. pegreffii* larvae might induce ribosomal stress in the early infection stage. However, the downstream pathways and post-infection responses require further study. Histopathology revealed severe inflammatory/hemorrhagic lesions caused by *Anisakis* infection in the rat stomach and muscle tissues in the first 32 h. The lesion sites showed infiltration by polymorphonuclear leukocytes (predominantly neutrophils and occasional eosinophils), and to a lesser extent, macrophages.

**Conclusion:** Understanding the cellular and molecular mechanisms underlying host responses to *Anisakis* infection is important to elucidate many aspects of the onset of anisakiasis, a disease of growing public health concern.

## Introduction

Tissue-dwelling intestinal nematodes encompass many diverse species, such as members of genera *Trichuris* (whipworms), *Strongyloides* (threadworms), *Necator, Ancylostoma* (hookworms), *Ascaris, Anisakis*, and *Trichinella*. Each species occupies a distinct host microenvironment, affecting, in turn, the type and effectiveness of the host immune response, the latter also being greatly influenced by the parasites' life cycle and behavior (1).

Typically, the host immune response to helminth infection is characterized by the T helper 2 (Th2) response, which involves the production of cytokines interleukin 3 (IL3), IL4, IL5, IL9, and IL13, eosinophilia, goblet, and mast cell hyperplasia, as well as alternatively activated macrophages (2). Despite the dominant Th2 phenotype, the central players in the course of helminth infection are naïve CD4^+^ T cells that can differentiate into several different regulatory and effector lineages (3). In addition to the pool of naïve T-cell precursors, the relatively plastic nature of Th subsets enables them to switch phenotype, mainly under the influence of varied cytokines (4). This ebb and flow of Th-cell populations in response to helminths has a major role in determining if the elicited immune kinetics will contribute to host protection or pathological onset of inflammation (3).

It is assumed that the immune response to tissue-dwelling intestinal nematodes fails to be modulated or down-regulated in an atypical or accidental host (5). An interesting experimental model of accidental infection in humans is rat anisakiasis caused by third-stage (L3) *A. pegreffii* larvae (6). In humans, L3 *Anisakis* larvae are either unable to molt to the subsequent stage (L4) and reach reproductive maturity or molting to L4 occurs extremely rarely (7). However, in the course of infection, patients can suffer significant clinical symptoms (8). The estimated frequency of anisakiasis in Japan is approximately 2000 cases/year and in South Korea 200 cases/year, while for some European countries estimates range from 20 (9) to 500 cases/year (10). However, a recently published quantitative risk assessment analysis estimated the risk of anisakiasis from the consumption of anchovies to be between 7700 and 8320 cases annually in the Spanish population (11). Thus, suggesting a considerable underestimation of the current number of cases in Europe. In addition, a multi-criteria decision analysis of food-borne parasites by European region ranked Anisakidae within the top 10 and top 5 in Northern and South-Western Europe, respectively (12).

Anisakiasis is caused by ingestion of live *Anisakis* L3 larvae in raw or undercooked seafood and it can develop according to the following scenarios: (i) expulsion of larvae with stool and/or vomit with no associated lesions of the gastrointestinal mucosa; (ii) attachment/embedding/penetration of larvae, most commonly affecting gastrointestinal tissues, or less commonly tissues encountered during larval migration through the peritoneal/pleural cavities, mesentery, liver, pancreas, lymph nodes, ovary, and subcutaneous tissues (13–15). Therefore, the disease is commonly classified as gastric, intestinal, and ectopic (or extra-gastrointestinal) (13, 14). In addition, ingestion of live *Anisakis* L3 can lead to sensitization and subsequent allergic reactions in humans. In a small number of cases, sensitized individuals may react even after ingestion of dead larvae (16). This clinical form is termed allergic/gastroallergic anisakiasis.

In a well-developed case of anisakiasis, fibrosis and eosinophilic infiltration into tissues surrounding the parasite are the most distinctive features of the local inflammatory response. Eosinophilic granulomas are observed in humans and infection models (17–20). Other cell lineages usually reported in histopathological examinations include mast cells, lymphocytes, and plasma cells (18). Additional component of the innate response includes Toll-like receptors (TLRs) on epithelial cells and activated dendritic cells (17).

To better understand accidental *Anisakis* infection in humans and subsequently develop effective intervention strategies, it is important to understand molecular and cellular mechanisms underlying host responses to this uncommon infection. Rodent models of intestinal nematode infection have proved relevant in mapping the cellular and molecular basis of mammalian protective mechanisms (21). In addition, previous research confirmed that *Anisakis* infection of rats leads to similar pathologies as humans (16). Therefore, the aim of this study was to: (i) describe histopathological changes at the infection site of *A. pegreffii* larvae in Sprague-Dawley rats; and (ii) qualitatively/quantitatively characterize the transcriptomes of affected tissues using RNA-Seq.

## Materials and methods

### Animal ethics

All animal experiments and protocols were approved by the Ethical Committee of the School of Medicine at the University of Split (registry number 2181-198-03-04-18-004), as well as the Veterinary and Food Safety Office of the Ministry of Agriculture (registry number 525-10/0255-16-7). Rat experiments were performed at the University of Split Animal Facility (permit number HR-POK-019) where they were raised and housed in pairs, in plastic cages with sawdust and corn bedding. The animals were kept in a controlled environment: food and water *ad libitum*, temperature 22 ± 1°C, with a 12 h light/dark cycle. The animals were separated in individual cages 24 h prior to the experiments and deprived of food 24 h before experimental infection.

### Larvae collection and rat infections

*A. pegreffii* larvae were collected from blue whiting *Micromesistius poutassou*, freshly caught in the C1 fishing zone of the Adriatic Sea (FAO 37.2.1), provided by a trusted dealer on the morning of the preliminary and experimental infection and delivered on ice. Gastric intubation and probe preparation protocols were adopted from the literature (20). Briefly, actively moving larvae were washed several times in physiological saline solution and checked under an Olympus BX 40 light microscope (Olympus Corp., Shinjuku, Tokyo, Japan) to confirm no cuticle damage had occurred during collection. Selected larvae were placed in previously prepared gastric probes. Each probe contained 10 larvae.

A preliminary experiment was performed to assess the temporal dynamics of *Anisakis* larvae infection. Samples were collected from 15 female Sprague-Dawley rats (average weight 197 ± 13.6 g, 3 animals per time point) at 6, 10, 24, 48, and 72 h post-infection.

Based on the results of the preliminary experiment, the duration of the *in vivo* experiment was set at 32 h post-infection. The experiment was performed on 35 male Sprague-Dawley rats in total (average weight 207 ± 20.1 g), with seven rats sacrificed per time point (6, 10, 18, 24, and 32 h post-infection). The experimental procedure remained the same.

Seven rats per group were administered a mixture of anesthetic and analgesic; Ketaminol (Richter Pharma AG, Wels, Austria), 50–100 mg/kg and Xylapan (Vetoquinol UK Ltd, Buckingham, UK), 5–10 mg/kg, by intraperitoneal injection, with additional administration of Ketaminol (Richter Pharma AG, Wels, Austria), 50–100 mg/kg, if toe pinch reflex was detected. Next, gastric intubation was performed with 10 *A. pegreffii* larvae (infected *N* = 5 rats per time point) or 1.5 ml of physiological saline solution (external control *N* = 2 rats). All animals were euthanized by decapitation, following an overdose of anesthesia by > 150 mg/kg Ketaminol (Richter Pharma AG, Wels, Austria) at designated time points. Tissue samples and recovered *A. pegreffii* larvae were collected, washed in physiological saline solution and immediately stored in Tri Reagent (Ambion Inc., Invitrogen, Carlsbad, CA, USA) at −80°C.

### Tissue preparation for semi-thin sections

For histopathological analysis, small fragments of the *Anisakis*-infected stomach (*N* = 5) and abdominal muscle tissues (*N* = 2) were collected and fixed in 4% paraformaldehyde in 0.1 M phosphate buffered saline (PBS) on ice. Tissue samples were post-fixed in 1% aqueous osmium tetroxide for 1 h, dehydrated in ascending series of acetone, and embedded in Durcupan resin (Honeywell-Fluka, Morris Plains, NJ, USA). Semi-thin 0.5 μm sections were cut, stained with 1% toluidine blue, and examined under an Olympus BX 40 light microscope (Olympus Corp., Shinjuku, Tokyo, Japan). Images were captured with an Olympus Camedia camera (Olympus Corp., Shinjuku, Tokyo, Japan) and assembled with Photoshop CS 5 software (Adobe Systems, San Jose, CA, USA).

### RNA and DNA extraction

Total RNA was extracted using Tri Reagent (Ambion Inc., Invitrogen, Carlsbad, CA, USA) following the manufacturer's protocol. Stomach and muscle tissues were selected when severe inflammatory/hemorrhagic lesions with or without migrating *Anisakis* larvae were observed. In addition, adjoining unaffected stomach and muscle tissues from the same rats were collected as the internal controls (referred to as unaffected tissues or control) and used in all statistical analyses for comparison with affected tissues. Details on stomach and muscle tissue samples are provided in Supplementary Table 1. Muscle tissues include abdominal, dorsal, intercostal muscle, and thoracic diaphragm. Stomach encompassed greater curvature, fundus, pyloric antrum, and pyloric canal. Hereafter, we will refer to our sequenced pools as “stomach” and “muscle” (tissues) when referring to RNA-Seq. In addition, RNA extraction was performed from the same tissues of uninfected rats (the external controls, referred to as uninfected, to avoid confusion with internal controls).

Additionally, DNA was extracted from 14 larvae found in the process of stomach/muscle penetration to confirm *Anisakis* species. DNA was extracted from Tri Reagent (Ambion Inc., Invitrogen, Carlsbad, CA, USA) after RNA extraction (data not shown) following the manufacturer's protocol and used for PCR-based restriction fragment length polymorphism (PCR-RFLP) analysis of the ribosomal DNA (rDNA) internal transcribed spacers (ITS-1 and ITS-2). RFLP pattern characteristic of *Anisakis simplex* (sensu stricto) × *A. pegreffii* putative hybrid (620-370-300-250 bp) (22) were observed for a single larva. All other larvae were confirmed as *A. pegreffii* according to an established RFLP pattern (370-300-250 bp) (22).

### cDNA preparation and illumina next-generation sequencing

Total RNA was dissolved in 20–40 μl of RNase/DNase free water (Merck Millipore, Billerica, MA, USA) and shipped on dry ice to the laboratory for advanced genomics at the Ruder Bošković Institute, Croatia, which provided the sequencing service. RNA concentration, purity, and integrity were determined using a 2100 BioAnalyzer (Agilent Technologies, Santa Clara, CA, USA) and Qubit 3.0 (Thermo Fisher Scientific, Waltham, MA, USA). Based on sample quality and specific lesion sites, 16 pools of at least three biological replicates, seven for muscle tissues and nine for stomach tissues, were created to obtain robust samples for estimation of gene expression changes depicting common signatures of affected vs. unaffected tissues (Supplementary Table 1). Following the manufacturer's protocol, the cDNA library was prepared using TruSeq Stranded mRNA kit (Illumina, San Diego, CA, USA) and subsequently sequenced using Illumina NextSeq 500 platform (Illumina, San Diego, CA, USA) over four lanes.

### RNA-seq raw reads pre-processing and mapping

The mean number of paired-end reads generated per sample was 31.2 million (range from 16.4 to 37.6 million reads). The quality assessment of the reads derived from different lanes using FASTQC (Babraham Bioinformatics, Babraham Institute, Cambridge, UK) indicated good data quality and no lane effects. Thus, the reads were joined into two paired FASTQ files per sample. Higher quality was observed for forward reads, as generally reported for Illumina sequencing (23). Trimmomatic (24) was used to trim Illumina adapter sequences, perform the sliding window clipping of reads (quality threshold 20, window size 4), and to remove reads shorter than 30 bases. Subsequently, PRINSEQ (25) was used to remove low-complexity reads (entropy threshold 70) and reads with more than 10% ambiguous (N) nucleotides. On average, 87% of paired-end reads survived this procedure and were mapped to *Rattus norvegicus* (v6) genome, Ensembl release 91 (26) using STAR (27). STAR (27) was also used to perform read counting. Approximately 90% of the reads were uniquely mapped. All data were submitted to NCBI Sequence Read Archive (SRA) under the accession number of SRP150499 (project ID PRJNA475982).

### Differential expression analyses

EdgeR (28, 29) package for R software ver. 3.4.2 (30), Bioconductor release 3.6 (31) was used for the count data exploratory analyses, log2-counts-per-million (logCPM) normalization, and inference of differential gene expression. Based on different sample variances and discrete clustering after principal component analyses (PCA), samples from stomach and muscle tissues were analyzed separately. Samples from uninfected rats were not included in statistical analyses, as they were represented by a single pool per dataset and were used only for comparative purposes in illustrations.

Low expression features (with less than one count per million per sample and at least three in any experimental condition) were filtered out prior to analysis. The dataset was mapped to Entrez Gene identifiers using rat annotation package org.Rn.eg.db (32). Statistical testing for differential expression was initially performed using a paired design with FDR cut-off of 0.05 and subsequently, relative to a minimum fold-change threshold of 2, using the edgeR implementation of *t*-tests relative to a threshold (TREAT) (33) method under the generalized linear framework. TREAT is an extension of the empirical Bayes moderated *t*-statistic presented by Smyth (34) and it achieves reliable *p*-values and FDRs for finding genes with differential expression that is biologically meaningful by including the fold-change threshold of interest in a formal hypothesis test. In this manner, we focused on genes with stronger regulation. Heatmap representations of the expression patterns of differentially regulated genes were plotted using ComplexHeatmap package (35).

### Enrichment analysis

The test for over-representation of Gene Ontology (GO) terms within differentially expressed genes (FDR < 0.05, |LogFC| >> 1) was conducted using edgeR's in-built goana function with FDR cut-off set at 0.05, taking into account gene length bias. The top 10 terms for stomach and top six for muscle associated with Biological Processes were selected and plotted using GOplot package (36) based on ggplot2 (37). The functional analysis of KEGG signaling, metabolic, and disease pathways (38) was conducted using GAGE (Generally Applicable Gene-set Enrichment), as implemented in the gage package (39). Log (base 2)-fold changes resulting after the linear model fit with edgeR were used as per gene score, and a two-sample *t*-test was run to compare mean log-fold changes *per set* relative to mean background log-fold change calculated using all filtered genes. Tests were run for up-, down-, and bi-directionally perturbed pathways using the default *q*-value cut-off of 0.1. Briefly, in results, we focused only on the metabolic and signaling sets with up- or downregulation that satisfied more stringent statistical significance criteria (*q*-value < 0.01). Pathview package (40) was used to visualize log-fold changes recorded for differentially expressed genes (FDR < 0.05) on their respective KEGG pathway maps, for pathways deemed significantly perturbed in GAGE analyses.

## Results

### Time course of infection

The preliminary experiment showed that the *A. pegreffii* infection duration (period from application of larvae until expulsion from the host) in rats was relatively short (Table 1). Six h post-infection, a fraction of the larvae had already left the host (between 80 and 90% were recovered from within the host in preliminary and experimental infections, respectively) and 24 h post-infection approximately 30% of applied L3 were found within the host. Larvae showed no synchronized migratory behavior correlated with time post-infection. Two larval clearance routes were observed. Route one was passage through the digestive tract facilitated by peristalsis with no apparent tissue damage. Route two was by penetration through the stomach, small intestine, caecum, or large intestine, with or without subsequent migration through the abdominal wall muscle. In a few cases, L3 larvae were found inside the pelvic cavity, both in preliminary and experimental infections. Epidermal penetration was also observed, but only in the preliminary experiment. In the majority of cases, L3 larvae were observed penetrating different parts of the stomach mucosa and abdominal muscles. Therefore, these tissues were the main subject of our downstream molecular and histopathological analyses.

**Table 1:** Experimental infection of Sprague-Dawley rats with third-stage *A. pegreffii* larvae (L3). Summary of the number of intubated animals, sampling time, organ, and percentage of recovered larvae.

**Test**	***N* animals**	**Sampling p/i (h)**	**% L3 recovered**	***SD* (%)**	**Organ of recovery (%)**
Preliminary	3	6	83.3	5.8	52% intestine, 36% stomach, 12% liver
Preliminary	3	10	55.6	41.1	35.3% stomach/intestine (each), 11.7% muscle, 5.9% epidermis/spleen/liver (each)
Preliminary	3	24	27.0	28.6	25% muscle/peritoneum/pelvic cavity (each) and 12.5% stomach/intestine (each)
Preliminary	3	48	16.7	15.3	100% stomach
Preliminary	3	72	15.0	21.2	33.3% peritoneum/spleen/intestine (each)
Experiment	5	6	89.3	13.6	46.7% stomach, 37.8% intestine, 6.7% peritoneum, 4.4% muscle, 2.2% liver/abdominal cavity (each)
Experiment	5	10	78.9	11.7	57% intestine, 38% stomach, 5% peritoneum
Experiment	5	18	38.3	33.3	70% stomach, 10% pelvic cavity/muscle/peritoneum (each)
Experiment	5	24	30.0	18.2	33.3% stomach, 25% muscle, 16.7% intestine/abdominal cavity (each), 8.3% peritoneum
Experiment	5	32	29.2	27.9	45.4% stomach, 27.3% muscle, 18.2% peritoneum, 9.1% intestine

*p/i, post-infection; SD, standard deviation*.

### Histopathology of *A. pegreffii* experimental infection

**Figure 1:**
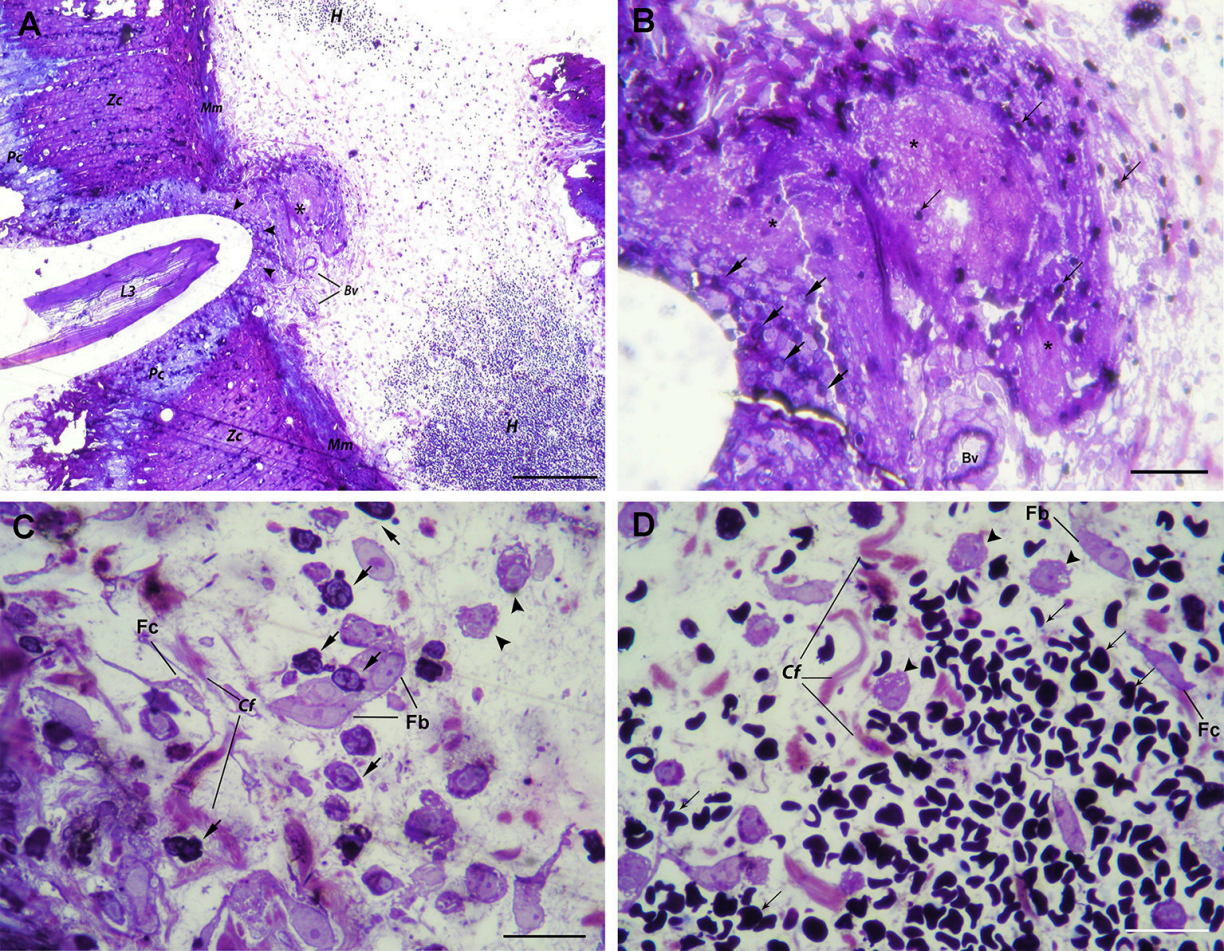
Histopathological findings in the stomach after *A. pegreffii* L3 larvae migration. (**A**) The early phase of migration of *A. pegreffii* L3 larvae through gastric wall mucosa causing compression and necrosis of surrounding parietal (Pc) and zymogenic cells (Zc). Larval migration caused rupture of both basement membrane and *muscularis mucosae* (Mm). A large necrotic area (asterisk) can be seen adjoining two blood vessels (Bv) right below the *muscularis mucosae*. Several strata of inflammatory cells (arrowheads) can be seen lining the site of larval penetration. At the periphery, extensive hemorrhage (H) with non-nucleated erythrocytes can be seen in the submucosa. Scale bar = 200 μm. **(B)** Detail of large necrotic area (asterisk) adjoining the blood vessel at the site of larval penetration, mixed with scant erythrocytes (thin arrows). At the bottom of lesion, neutrophils (arrows) can be seen lining the site of larval penetration. Scale bar = 50 μm **(C)** High magnification of inflammatory infiltrate closer to the site of penetration and large necrotic area with numerous neutrophils (arrows) and occasional macrophages (arrowheads) interspersed between collagen fibers (Cf), fibroblasts (Fb), and fibrocytes (Fc). Scale bar = 20 μm. **(D)** High magnification of extensive submucosal hemorrhage with numerous non-nucleated erythrocytes (thin arrows) and several macrophages (arrow heads) interspersed between collagen fibers (Cf), occasional fibroblasts (Fb), and fibrocytes (Fc). Scale bar = 20 μm (1% toluidine blue).

In the stomach, *A. pegreffii* larvae were found in the early phase of migration through the gastric wall, causing compression and necrosis of surrounding parietal and zymogenic cells in the mucosa (Figure 1A). Several strata of polymorphonuclear leukocytes (predominantly neutrophils and occasional eosinophils), and to a lesser extent, macrophages, were observed infiltrating penetration site. However, these cells appeared not to be in the direct contact with larval cuticle, probably due to tissue shrinkage during sample processing. Moreover, the rupture of both basement membrane and *muscularis mucosae* occurred at the site of larval migration. Close to the two adjoining arteries in the submucosa, a large necrotic area admixed with scant inflammatory infiltrate and mild hemorrhage was observed (Figures 1A,B, asterisk). However, extensive hemorrhages, visible at gross pathological examination, were also noticeable in the submucosal layer. The submucosa was predominantly infiltrated with polymorphonuclear leukocytes (predominantly neutrophils), and macrophages, interspersed between connective tissue fibers, fibroblasts, and fibrocytes. Extravasated polymorphonuclear leukocytes concentrated toward the necrotic area adjoining blood vessels and the migrating larva (Figure 1C). Macrophages were seen in deeper submucosa strata, closer to the large submucosal hemorrhage (Figure 1D).

**Figure 2:**
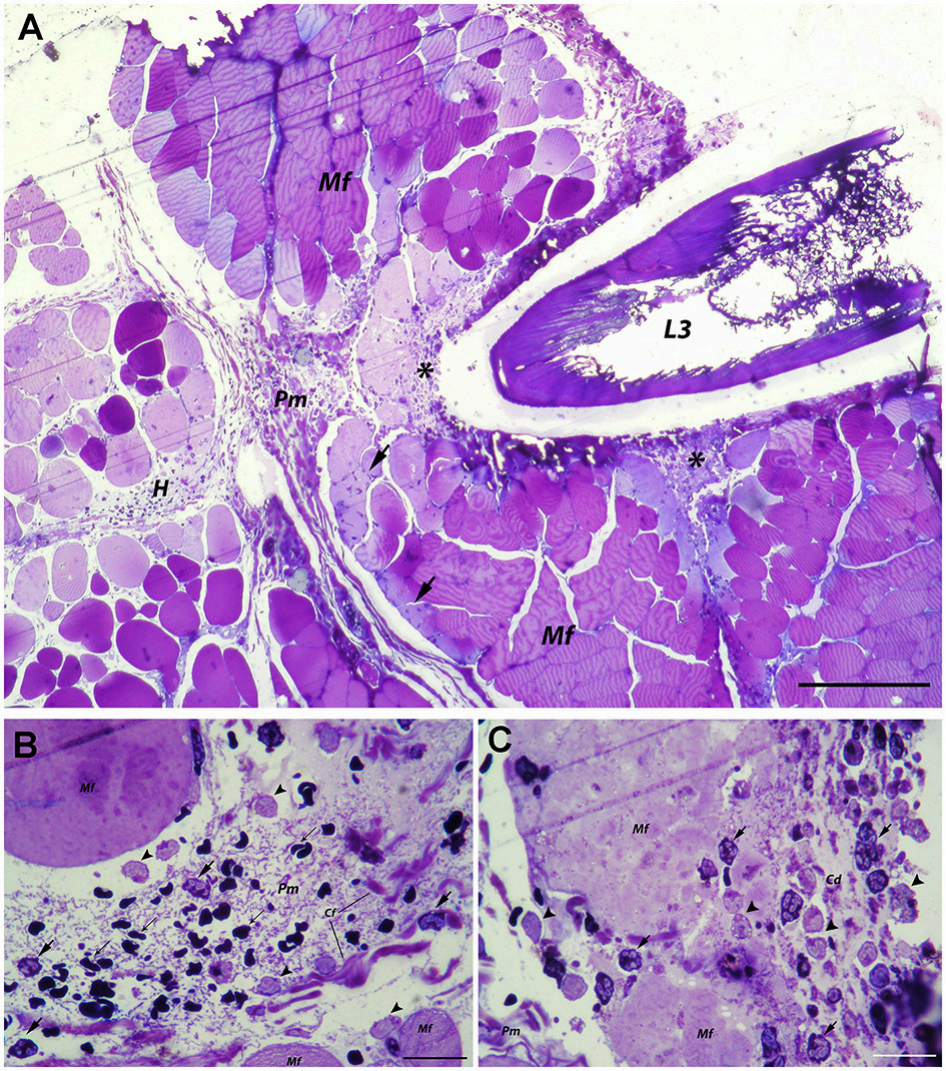
Histopathological findings in abdominal muscle after migration of *A. pegreffii* L3 larvae. **(A)** Migration of *A. pegreffii* L3 larvae through perimysial connective tissue causing tissue necrosis and structural disintegration of muscle fibers (Mf) along the migration path. Large areas of cellular debris mixed with abundant inflammatory infiltrate (asterisks) can be seen. Moderate hemorrhage (H) in perimysium (Pm) together with moderate inflammatory infiltrate can be seen in close proximity to the larva. Occasional polymorphonuclear leukocytes can be seen in endomysium and migrating over muscle fibers (Mf) toward the site of larval penetration (arrows). Scale bar = 200 μm, **(B)** High magnification of perimysial hemorrhage (H) with non-nucleated erythrocytes (thin arrows) and inflammatory infiltrate composed mostly of neutrophils (arrows) and scant macrophages (arrowheads) interspersed between connective tissue fibers. **(C)** High magnification of necrotic area showing the structural disintegration of affected muscle fibers (Mf) and abundant cellular debris (Cd) mixed with numerous neutrophils (arrows) and macrophages (arrowheads) close to the larva in the perimysium (Pm; 1% toluidine blue). Scale bar = 20 μm.

In the abdominal musculature, penetrating *A. pegreffii* larva caused tissue fragmentation and necrosis along its migration path (Figure 2A). The perimysial connective tissue exhibited extracellular edema and moderate hemorrhage with inflammatory infiltration, the latter composed of polymorphonuclear leukocytes (predominantly neutrophils), and macrophages (Figure 2B). A few polymorphonuclear leukocytes were also seen in the endomysium or between myocytes at the site of larval penetration. Higher magnification revealed structural disintegration and necrosis of muscle fibers surrounding the larva, with an accumulation of abundant cellular debris mixed with numerous polymorphonuclear leukocytes and macrophages (Figure 2C).

### Differentially expressed genes

**Figure 3:**
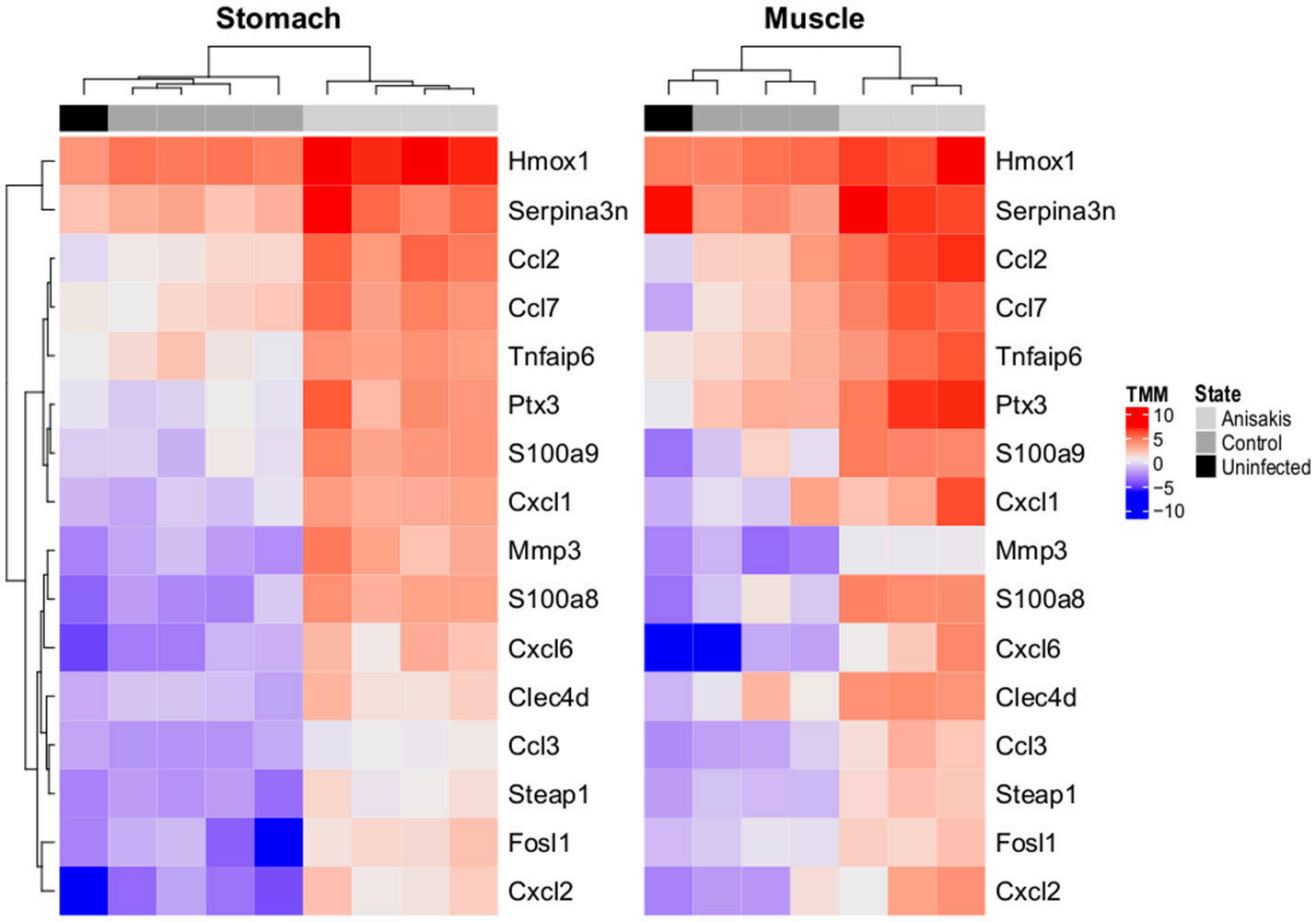
Two-dimensional hierarchical clustering and heatmap display of log2-counts-per-million (logCPM)-normalized expression values of differentially expressed genes common (*N* = 16) in rat stomach and muscle penetrated by *A. pegreffii* larvae, adjoining unaffected tissues of experimentally infected Sprague-Dawley rats (Control), and the same tissues of uninfected rats (Uninfected, animals intubated with physiological saline solution). Genes are clustered according to profiles observed in stomach.

Consistent with the non-synchronized migrating behavior of L3 larvae during the experiment and no preference for penetration site within inspected tissues; somewhat higher variability was observed between samples within experimental groups using PCA (Supplementary Figure 1). Nevertheless, statistical analysis detected common gene regulation patterns associated with *Anisakis* infection in the stomach and muscle tissues. In total, there were 1372 (855 up and 517 down) differentially expressed (DE) genes (FDR < 0.05) in the *Anisakis*-infected stomach tissues, and 1633 (1230 up and 403 down) DE genes in the muscle tissues of rats (Supplementary Figure 2 and Supplementary Table 2), in comparison with their respective matched controls. Only a fraction of these upregulated DE genes passed the TREAT test and demonstrated strong and significant fold changes (LogFC >> 1): 41 in the stomach and 111 in the muscle tissues. Among the genes with the greatest logFC, 16 were common for the infected stomach and muscle tissues (Figure 3). The expression profiles of the uninfected samples for both datasets were consistent with control samples (Figure 3). These include S100 proteins, namely *S100a8* (4.68 logFC in stomach and 4.62 in muscle) and *S100a9* (3.53 logFC in stomach and 4.41 in muscle). S100A8 is a calcium- and zinc-binding protein with an important role in the regulation of inflammatory processes and the immune response and is also involved in orchestrating chemotaxis and adhesion of neutrophils. S100A8/A9 is predominantly present as calprotectin, displaying a plethora of intra- and extracellular functions. S100A8/A9 proinflammatory activity includes promotion of cytokine and chemokine production, and evidence for this activity can be supported by a number of expressed *Ccl* (*Ccl2, Ccl3*, and *Ccl7*) and *Cxcl* (*Cxcl1, Cxcl2*, and *Cxcl6*) chemokines (Figure 3) in *Anisakis*-infected tissues. Another highly expressed gene in both stomach and muscle tissues was matrix metallopeptidase 3 (*Mmp3*, 4.43 logFC in stomach and 2.89in muscle), which is involved in degradation of fibronectin, laminin, collagens and cartilage proteoglycans, and activation of procollagenase.

### Functional interpretation of transcriptomic changes induced by *anisakis* infection

**Figure 4:**
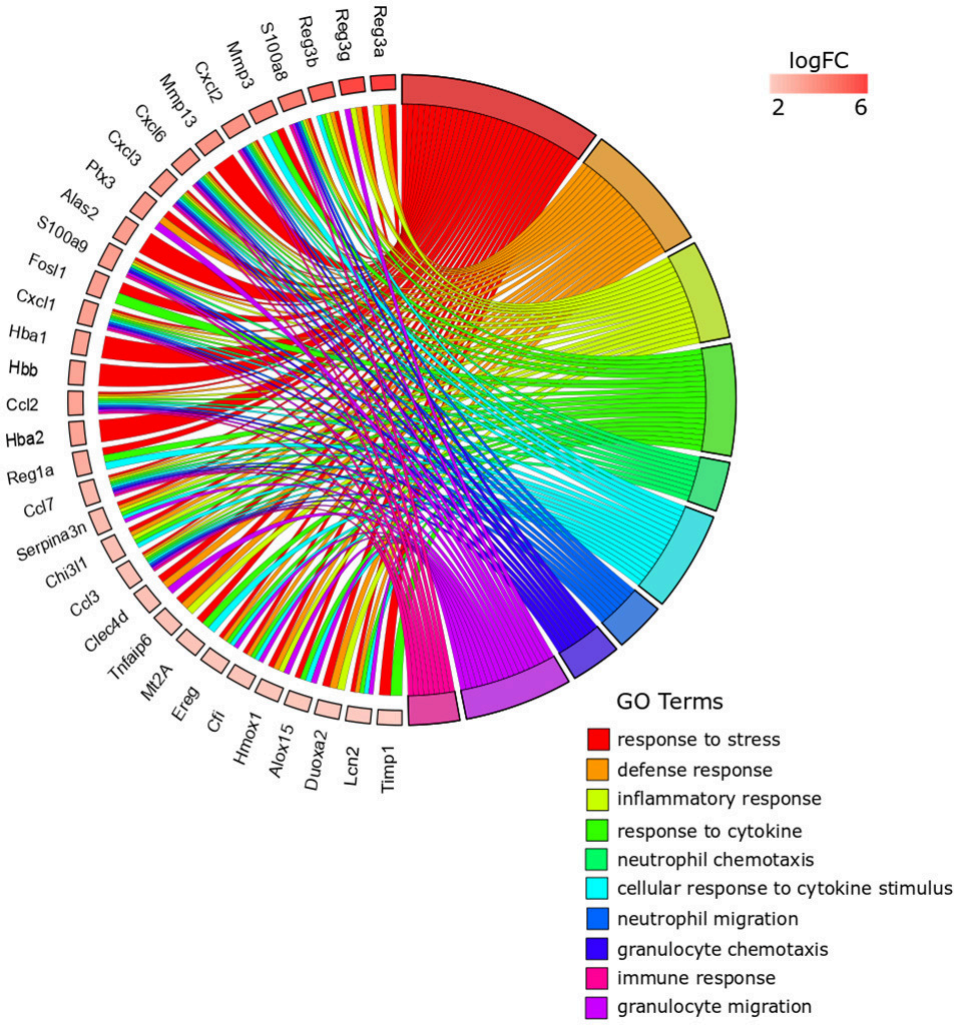
GOChord plot of top 10 ranked overrepresented GO terms belonging to the Biological Process subontology for *Anisakis*-infected rat stomach. The genes are linked to their assigned terms via colored ribbons. Genes are ordered according to the observed log-fold change (logFC), which is displayed in descending intensity of red squares displayed next to the selected genes.

From the 259 overrepresented GO terms (FDR < 0.05) associated with the 41 top DE genes for *Anisakis*-infected rat stomach, the top five ranked were response to stress, defense response, inflammatory response, response to cytokine, and neutrophil chemotaxis (Figure 4, Supplementary Table 3). The presence of hemoglobin-related genes [hemoglobin alpha 1 (*Hba1*) and 2 (*Hba2*) and hemoglobin subunit beta-1 (*Hbb*)] was directly related to response to stress. Several regenerative family member genes (*Reg3a, Reg3g, Reg3b*, and *Reg1a*), with a particularly pronounced upregulation in stomach (Figure 4 and Supplementary Table 2), were also associated with other inflammation, immune, and defense-related sets. The remainder of the top five GO terms had many genes in common. In addition to *S100a8/S100a9* and *Ccl/Cxcl* chemokines, interesting transcripts encompassed lipocalin 2 (*Lcn2*), involved in multiple processes such as apoptosis and innate immunity, and chitinase 3-like 1 (*Chi3l1*), which plays a role in Th2 inflammatory response and IL13-induced inflammation.

**Figure 5:**
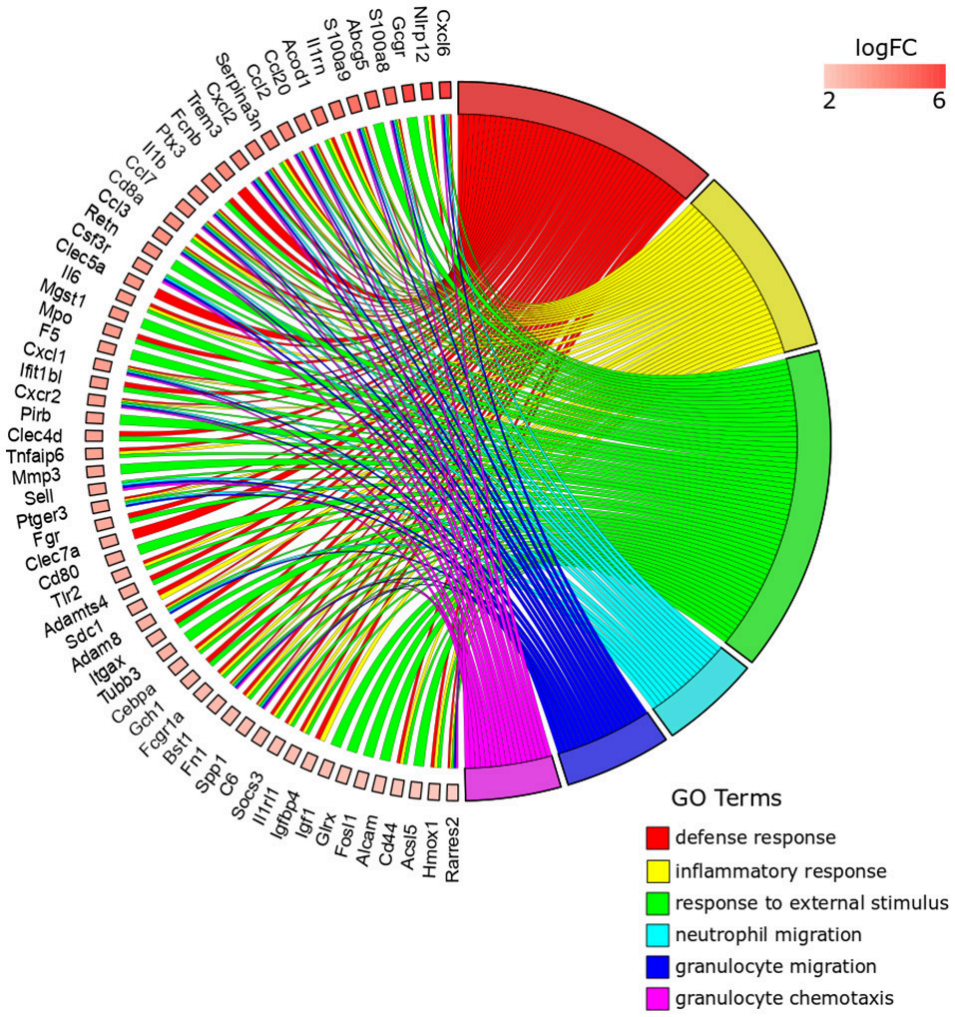
GOChord plot of top six ranked overrepresented GO terms belonging to the Biological Process subontology for *Anisakis*-infected rat muscle. The genes are linked to their assigned terms via colored ribbons. Genes are ordered according to the observed log-fold change (logFC), which is displayed in descending intensity of red squares displayed next to the selected genes.

The top five ranked overrepresented GO terms for the *Anisakis*-infected rat muscle, out of 494 selected (FDR < 0.05), were very similar to the stomach, but ranked differently: defense response, inflammatory response, response to external stimulus, granulocyte migration, and neutrophil migration (Figure 5 and Supplementary Table 3). The response to stress was also found in the overrepresented GO terms list for the muscle, although ranked 21st according to FDR (Supplementary Table 3). The largest set of DE genes was found in the response to external stimulus, followed by the defense response (Figure 5). Some of the genes were associated only with the response to external stimulus term, such as activated leukocyte cell adhesion molecule (*Alcam*), which promotes T-cell activation and proliferation via its interactions with Cd6. However, most of the genes were common to the top five GO terms. In addition to the omnipresent *S100a8/S100a9* and *Ccl/Cxcl* chemokines, two other potent pro-inflammatory cytokines are worth mentioning; interleukin 1β (*Il1*β) and *Il6*, the latter known as the inducer of the acute phase response.

**Figure 6:**
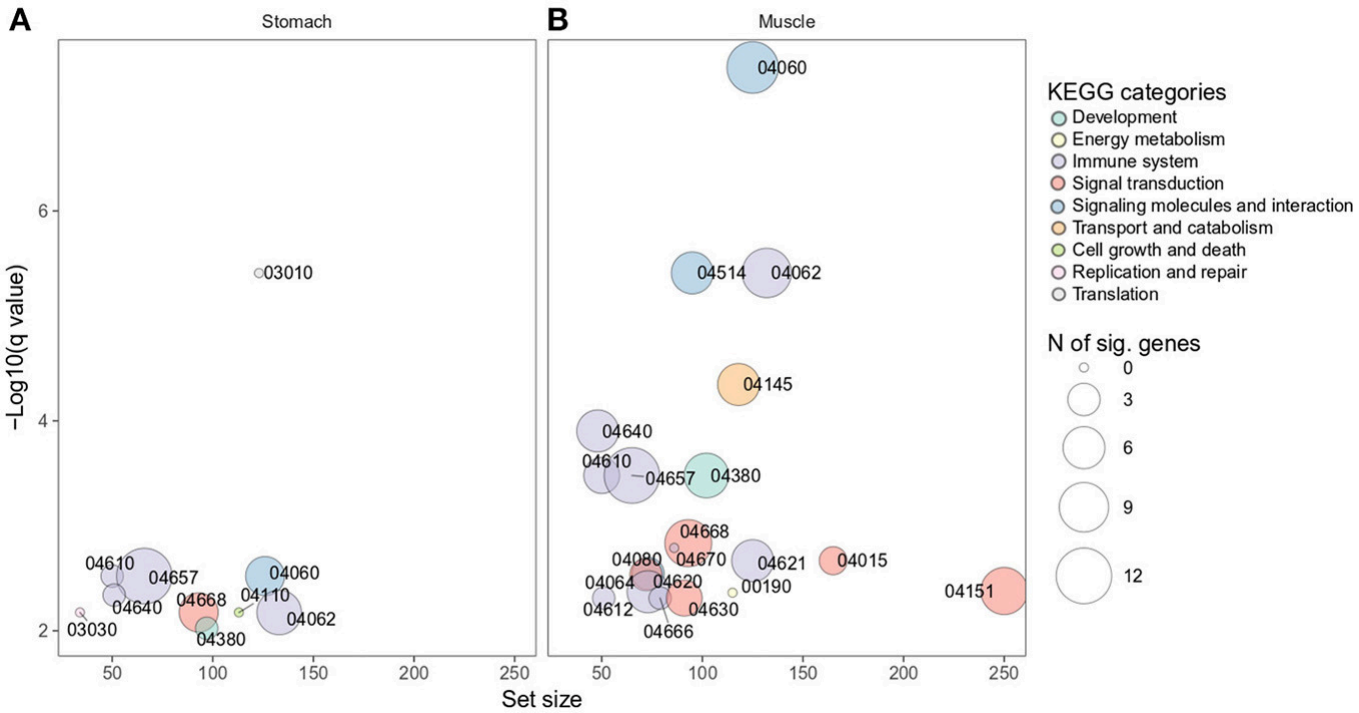
Overrepresented KEGG metabolic and signaling pathways differentially perturbed in rat stomach **(A)** and muscle **(B)** infected with *A. pegreffii*. Set size is plotted against log10 *q*-value and circle size depicts the number of DE genes per each pathway. 04060 Cytokine-cytokine receptor interaction, 04062 Chemokine signaling pathway, 04514 Cell adhesion molecules (CAMs), 04145 Phagosome, 04640 Hematopoietic cell lineage, 04610 Complement and coagulation cascades, 04657 IL17 signaling pathway, 04380 Osteoclast differentiation, 04668 TNF signaling pathway, 04670 Leukocyte transendothelial migration, 04015 Rap1 signaling pathway, 04621 NOD-like receptor signaling pathway, 04080 Neuroactive ligand-receptor interaction, 04064 NF-κB signaling pathway, 04151 PI3K-Akt signaling pathway, 04620 Toll-like receptor signaling pathway, 04666 Fc gamma R-mediated phagocytosis, 04612 Antigen processing and presentation, 04630 Jak-STAT signaling pathway, 00190 Oxidative phosphorylation, 03010 Ribosome, 03030 DNA replication, 04110 Cell cycle.

**Figure 7:**
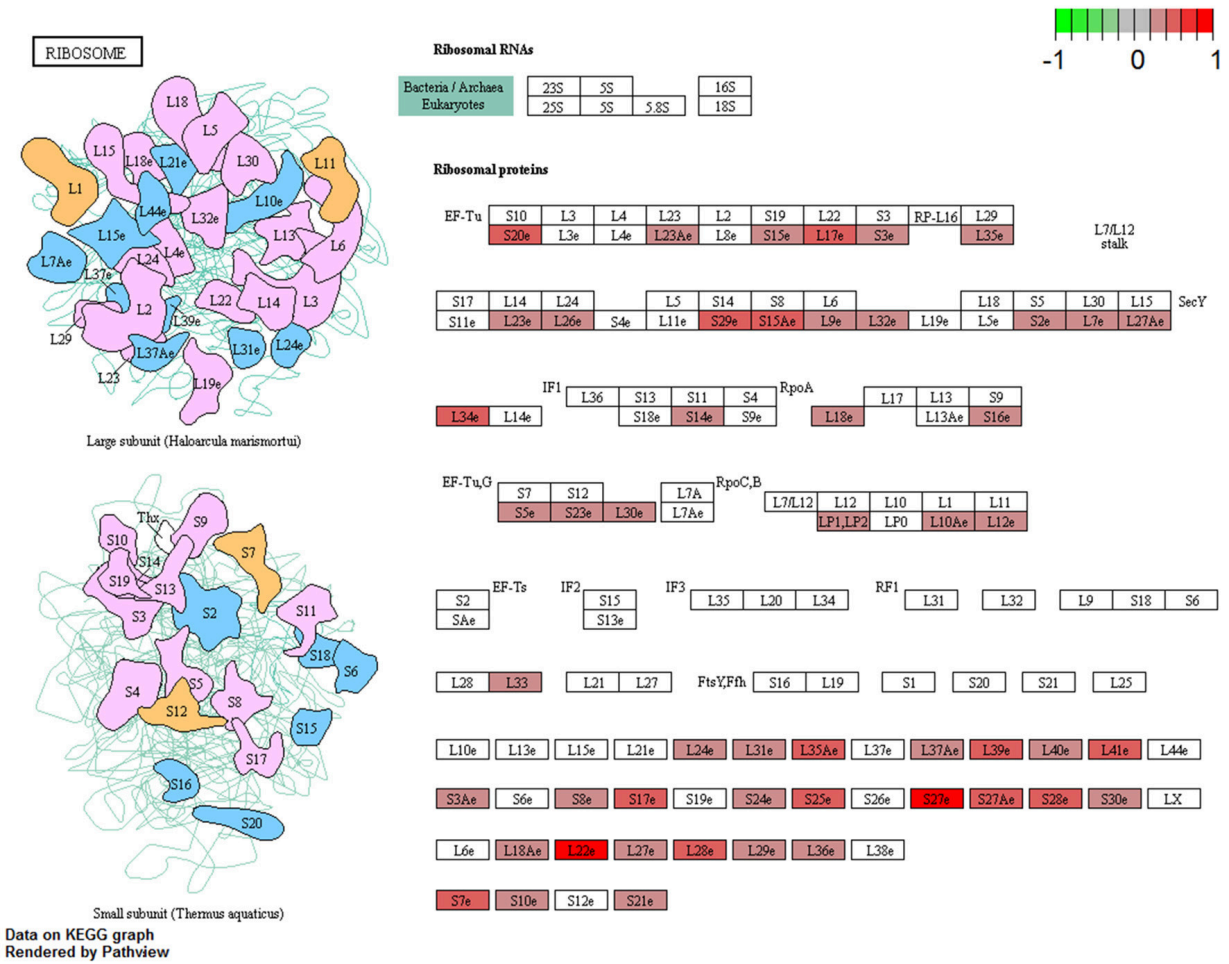
Visualization of KEGG Ribosome pathway genes differentially perturbed (FDR < 0.05) in rat stomach infected with *A. pegreffii*. Plotted using Pathview package for R/Bioconductor.

**Figure 8:**
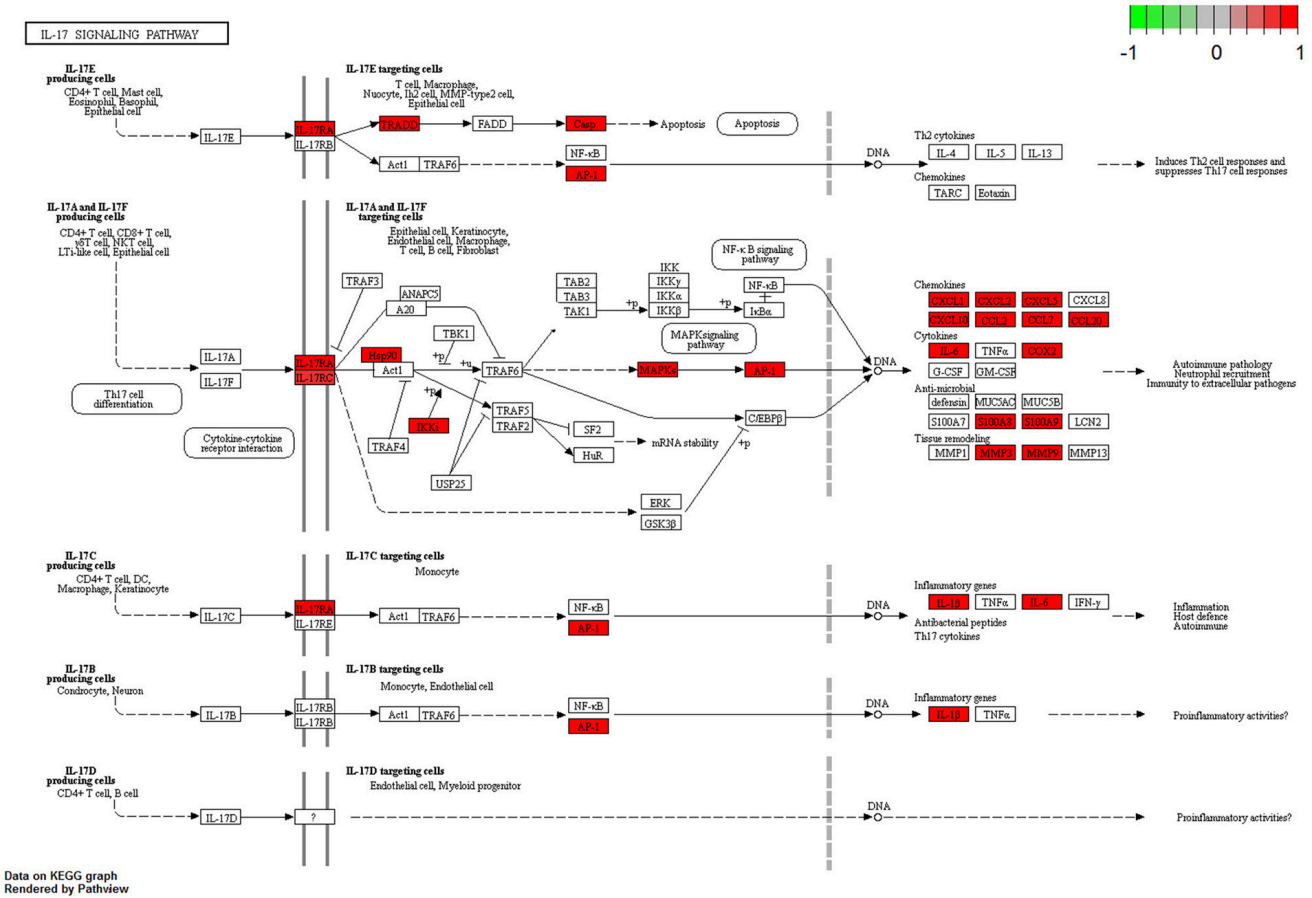
Visualization of KEGG IL17 signaling pathway genes differentially perturbed (FDR < 0.05) in rat muscle infected with *A. pegreffii*. Plotted using Pathview package for R/Bioconductor.

The complete KEGG pathway analysis results are shown in Supplementary Table 4, including metabolic, signaling, and disease sets for the infected stomach and muscle tissues undergoing *Anisakis* larval migration. Metabolic and signaling sets with up- or downregulation that satisfied more stringent statistical significance criteria (*q*-value < 0.01) are shown in Figure 6. The top five overrepresented KEGG metabolic and signaling pathways upregulated in the rat stomach included 03010 Ribosome, 04610 Complement and coagulation cascades, 04060 Cytokine-cytokine receptor interaction, 04657 IL17 signaling pathway and 04640 Hematopoietic cell lineage (Figure 6 and Supplementary Table 4). Except for 03010 Ribosome (Figure 7), the rest of the top five KEGGs were also found in the infected muscle, however, not all appeared in the top five list (Supplementary Table 4). In addition to, 04060 Cytokine-cytokine receptor interaction and 04640 Hematopoietic cell lineage, the top five upregulated KEGG pathways for rat muscle included 04062 Chemokine signaling pathway, 04514 Cell adhesion molecules (CAMs), and 04145 Phagosome (Figure 6 and Supplementary Table 4). The only downregulated KEGG pathway in the rat muscle was 00190 Oxidative phosphorylation. According to KEGG functional categories, the greatest number of differentially perturbed pathways belongs to the immune system, followed by signal transduction and signaling molecules and interaction (Figure 6). The pathways with the greatest number of DE genes (*N* = 12) included in both the stomach and muscle were 04657 IL17 signaling pathway (Figures 6, 8) and 04060 Cytokine-cytokine receptor interaction (*N* = 10). 04657 IL17 signaling pathway is shown only for muscle data (Figure 8), as there were only slight differences between the two datasets.

## Discussion

This is the first RNA-Seq transcriptomic analysis of vertebrate host tissues affected by *Anisakis* larval migration. We chose Sprague-Dawley rats as the host model to simulate the course and pathogenesis of accidental infection in humans. In addition, molecular identification of the anisakid confirmed that *A. pegreffii* was the parasite in our study, which is important to consider, as it is the most frequent causative agent of anisakiasis in Mediterranean countries (18, 19, 41, 42). An *in vivo* and *in vitro* study in the same animal model confirmed the pathogenic potential of *A. pegreffii* The study also found that *A. pegreffii* larvae tend to remain in the stomach of rats much longer than *Anisakis simplex* (sensu stricto) (6). Consistent with our study, the authors concluded that the migratory behavior of L3 larvae was non-synchronous and L3 larvae showed no preference for penetration site within inspected tissues. However, the authors did not report muscle penetration, probably due to the shorter time-span of the experiment (24 h) (6). While RNA-Seq has previously been applied to identify and characterize putative novel parasite allergens in *Anisakis* (43) and to reveal the pathogenic mechanisms associated with infection in two genus members, *A. simplex* (s. s.) and *A. pegreffii* (44), no description of the host transcriptomic response has yet been given.

Even though anisakids are not natural human parasites and they are not evolutionary adapted to each other (5), both human and murine models of *Anisakis* infection have been predominantly characterized in the context of Th1, Th2, or Th1/Th2 immune responses inferred through different serological studies aimed at defining the kinetics of allergic reactions to the nematode after different sensitization regimes (16, 45, 46). By assessing immune response traits in L3-infected *Anisakis-*naïve rats, we have identified an acute proinflammatory host response, which seems to be predominantly characterized by the activation of a Th17 lineage of effector T helper lymphocytes. This lineage is known for the production of IL17 (47), a cytokine that induces secretion of chemokines and antimicrobial peptides in diverse tissues and cell types (fibroblasts, endothelial cells, epithelial cells, keratinocytes, and macrophages), consequently leading to the recruitment of neutrophils and an enhanced proinflammatory reaction (48). Indeed, we found both neutrophils and macrophages mixed with necrotic cells and cellular debris in our study. Necrosis can stimulate inflammation due to leakage of intracellular components into surrounding tissue (49). Such a scenario can be more pronounced in tissues with high metabolic activity, such as the stomach epithelium, which is rich with parietal and zymogenic cells producing hydrochloric acid and various digestive enzymes, respectively (50). Necrosis and eosinophils are usually present in the center of granulomas caused by *Anisakis* larval remains, together with foreign-body giant cells and connective tissue. Subsequently, the cellular infiltration encompasses primarily lymphocytes, as memory T and B cells are generated during primary infection of the host. Thus sensitized, hosts can experience allergic reactions upon re-exposure to *Anisakis* (16).

In previous allergy models of *Anisakis* infection in C57BL/6 mice, significantly increased Th17-related cytokines IL6 and IL17A were observed after subchronic treatment (7 times over 15 days) with recombinant Ani s 1 (rAni s 1) allergen (51). *In vivo* results were further supported by proliferation and activation of IL6 and IL17A secretion in splenocytes treated with rAni s 1 or rAni s 9, suggesting that IL17 might play a critical role in the *Anisakis*-associated allergic reaction (51). However, in another study, Wistar rats exposed orally to fresh and frozen *Anisakis* larvae (treated twice over 7 days and sampled after 21 days) had a significantly higher level of plasma IL17. Notably, intraperitoneal injection of crude larval extract did not induce significant IL17 production (52). This might suggest that live larvae trigger different immune recognition and activation pathways than crude extracts or even secretory/excretory products (53). This hypothesis is supported by a recent study where dendritic cell (DC) cytokine and chemokine secretion was conditioned by live *A. pegreffii* larvae or crude extract (54). Interestingly, the autologous CD4^+^ T cells that were stimulated by DCs differentiated in the presence of live larvae or crude extract but failed to produce IL4, IL17, and IL10. Observing no IL17 production in both scenarios, the authors concluded that DCs may contribute to establishing localized inflammation at the earliest stage of infection, but their role in Th2/Th17 or T regulatory (Treg) polarization remains inconclusive. Similarly, Th17 and IL17 signaling pathways seem to be strongly induced in our study, without regulation of the *Il17* gene.

Conversely, Th17 cells are known to produce cytokines that are specialized in promoting responses against extracellular bacteria and fungi (55). However, *Trichinella spiralis*, another zoonotic nematode suggested as the best terrestrial counterpart of the tissue-dwelling marine *Anisakis*, can elicit both Th2- and Th17-related immune responses during its intestinal phase, which occurs after the first week of infection (56). The life cycle of *T. spiralis* in humans begins with the consumption of meat containing the first-stage muscle larvae. After larval release from the cyst and penetration into epithelial cells of the small intestine, the intestinal phase begins, where larvae molt, grow and reproduce (57). However, at the onset of nurse-cell formation (induced by the parasite approximately 1-week post-infection), Th2 and Th17 responses are inhibited by Treg cell recruitment to the peripheral lymph nodes near the nurse cells 2-weeks post-infection (56). This shows that Th2 and Th17 play crucial roles in the initiation of the inflammatory response to nematode infection, as observed in our study, which can subsequently develop in another direction as infection becomes chronic.

Interestingly, we found upregulated diseases-related KEGG pathways in the rat stomach and muscle that are principally associated with strong inflammatory responses, such as 05323 Rheumatoid arthritis and 05321 Inflammatory bowel disease (Supplementary Table 4), corroborating the observed Th17 response in experimental *Anisakis* infection. As Th17 cells are potent inducers of tissue inflammation, they are also associated with the pathogenesis of many autoimmune diseases and human inflammatory conditions. In experimental animal models, Th17 cells are responsible for autoimmune encephalitis, collagen-induced arthritis, colitis, and psoriasis (58–60). Furthermore, the numerous bacteria-related diseases significantly upregulated in our stomach and muscle dataset, such as rno05150 *Staphylococcus aureus* infection and rno05132 *Salmonella* infection (Supplementary Table 4), are most likely due to the extensive study of the Th17 response in bacterial infections.

The frequent appearance of the bacteria-related host immune responses in our results posits another possible explanation. The host intestinal mucosal immune system is a unique environment that must restrain immune responses directed against commensal microbes and dietary antigens while also protecting mucosal tissue during nematode parasite infection (1). It appears that commensal microbiota are physically separated from intestinal surfaces and this barrier is critical for limiting immune activation and maintaining homeostasis and mutualistic host-microbe associations (61). Furthermore, perturbed spatial relationships between microbiota and host correlate with disease states such as inflammatory bowel disease (62). RegIIIγ, a secreted antibacterial lectin that we identified as the top DE gene in the rat stomach tissues (logFC 6.25), is essential for maintaining a ~50 μm zone that physically separates the microbiota from the small intestine epithelium (63). In addition, loss of the host-bacterial segregation in RegIIIγ^−/−^ mice results in increased bacterial colonization of the intestinal epithelium and activation of intestinal adaptive immune responses by the microbiota (63). It is plausible that the tissue damage induced by *Anisakis* penetration into the stomach mucosa disrupted the delicate host-microbe microenvironment, resulting in the host immune response being targeted toward bacteria crossing the mucosal barriers, whether of autochthonous origin or carried over by contact with *Anisakis* larvae.

Another important inflammation-related pathway upregulated in our study is the KEGG 04657 IL17 signaling pathway, implicated by 12 DE genes in both the stomach and muscle tissues of rats. IL17A and IL17F have protective roles in the host mucosal barrier defense mechanism against certain pathogens. Although mostly related to bacterial infections (64), they have also been associated with filarial nematodes (65) as well as other nematode infections (66). Both stomach and muscle datasets had S100 proteins (*S100a8* and *S100a9*), matrix metallopeptidase (*Mmp3* and *Mmp13*), and Cxcl and Ccl chemokines (*Cxcl1, Cxcl2, Ccl2, Ccl7*, and *Ccl20*) from the IL17 signaling pathway. *Il6* was present only in the muscle data and lipocalin 2 (*Lcn2*) was present only in the stomach dataset. S100A8/A9 proinflammatory functions were manifested through leukocyte recruitment, increased cytokine and chemokine production, and regulation of leukocyte adhesion and migration in our study (67). Nematode infections have previously been reported as the reason behind neutrophil recruitment in response to high S100A8 and S100A9 protein expression levels. For example, neutrophil recruitment was significant in a transient lung inflammatory response caused by the migratory phase of *Litomosoides sigmodontis* filarial infective larvae in BALB/c mice (68). The intracellular and extracellular functions of *S100a8/a9* in the rat stomach and muscle were further supported by observation of neutrophil/leukocyte chemotaxis and migration, cytokine and chemokine production, upregulation of TLR and NF-κB signaling pathways (67), supporting an intensive inflammatory onset timed by 32 h post*-*infection in our study.

Interestingly, no evidence to support active wound healing was found within the transcriptomes of stomach and muscle tissues. However, it is possible that among the myriad MMPs that appeared in the DE stomach and muscle tissue gene lists (*Mmp3, Mmp10, Mmp13*, and *Mmp19*, to name some of the most significant), some were involved in tissue remodeling and repair in response to the injury (69). We abstain from inferring a generalized assumption on the subject, as a specific MMP secreted by one cell type (for example, a macrophage) probably performs a different function than the same enzyme produced by another cell type (for example, an epithelial cell) (70). However, MMP functions encompass inflammation and immunity, acting on pro-inflammatory cytokines, chemokines, and other proteins (70). In addition, it is believed that MMP13 plays a role in wound healing via a mechanism that probably involves activation of transforming growth factor beta 1 (TGFβ1) and degradation of connective tissue growth factor (CTGF), as well as in keratinocyte migration during wound healing (67). In our study, however, we believe that hemorrhagic lesions and IL1β and IL6 could have delayed the wound healing process (71) or the sampling occurred at too early in the process to fully capture it. Similarly, the appearance of fewer eosinophils than neutrophils is most likely the result of sampling in the peracute phase, which occurs prior to the typical eosinophilic infiltration of the tissue surrounding the parasite and before the Th2 response. According to the literature, eosinophilia does not typically develop until several days after the onset of clinical symptoms in anisakiasis (72).

Furthermore, we observed an intensive neutrophil and macrophage infiltration into the damaged tissues, which may have potentially biased consequences. Initially, their beneficial action results in removing the infectious agent, clearing debris, and expressing factors that promote wound healing. However, during the healing process, the control of inflammation may be crucial for effective tissue repair (73), with macrophages having an important role in the resolution of tissue damage, as observed in the course of viral infection (74). In addition, IL17 may also indirectly contribute to the early stages of the tissue repair process, as it serves as a mediator of successive neutrophil recruitment and activation (75). However, prolonged production of the IL17 also potentiates inflammation and tissue damage (76).

IL6 might be an important factor in *Anisakis* epidemiology (51) and it appeared in several differentially perturbed pathways in our study. In addition to its role in activation and differentiation of macrophages, lymphocytes, and terminal differentiation of B cells, IL6 also regulates acute and chronic inflammation (77). Murine studies have identified TGFβ and IL6 as key cytokines driving Th17 differentiation (78, 79). Additionally, pro-inflammatory cytokines such as IL1β and tumor necrosis factor α (TNFα) can increase the efficiency of Th17 differentiation (80). Both *Il6* and *Il1*β were DE in the *Anisakis*-infected rat muscle. However, the role of *Il6* in our study should be interpreted with caution, as IL6 can also act as an anti-inflammatory myokine, which is elevated and discharged into the bloodstream in response to the muscle contraction (81). Hyperactivation of IL6/STAT3 signaling in the muscle may stimulate the synthesis and systemic release of the acute-phase response proteins such as serum amyloid A (SAA) and fibrinogen, which may amplify catabolic signals in the muscle, turning muscle into a key player in innate immunity in experimental cancer-induced muscle wasting in mice (82). Whether *Anisakis* infection triggers pro- or anti-inflammatory IL6 responses remains to be elucidated.

In addition to the remarkable proinflammatory response based on Th17 and IL17 signaling pathways and DE genes included in these pathways, we also observed the stress response. The highest-ranking GO term in rat stomach was the response to stress, but the strongest *q* value was unexpectedly designated to the KEGG 03010 Ribosome pathway. This feature was specific to *Anisakis*-infected stomach. Although none of the ribosomal genes in our study showed large expression differences, they were found consistently upregulated (FDR < 0.05) across the stomach dataset (Supplementary Table 2). A visualization of the KEGG Ribosome pathway differentially perturbed in the *Anisakis*-infected rat stomach is shown in Figure 7. This perturbation suggests that *Anisakis* infection could have triggered the onset of ribosomal stress during penetration of the stomach mucosa. Interestingly, most ribosomal proteins have been implicated in host immune responses by boosting immune signaling or facilitating pathogen proliferation under various circumstances (83).

In the host-parasite context, ribosomal stress has only been addressed in a single study of pig infection with *Cryptosporidium parvum* merozoites (early asexual stages), where both host and parasite responses were assessed by RNA-Seq. Interestingly, transcripts of the *C. parvum-*infected pig intestinal cells did not reveal stress- and apoptosis-related genes. However, genes encoding ribosomal functions were highly enriched in both host and *C. parvum* cells, although to a greater extent in the parasite than the host (84). The authors pointed out that transcriptomic changes observed might be influenced by rapid parasite proliferation forcing a high energetic and metabolic load on host cells. However, they did not associate it directly with ribosomal genes. Supported by the number of DE ribosomal genes in the *Anisakis*-infected rat stomach tissues and in light of their recently discovered activities in host immune responses, we argue that *A. pegreffii* larvae could induce such a response at the early infection stage in specific tissues. The downstream pathways and whether it is eventually balanced in the post-infection stages remains to be elucidated.

## Conclusion

Our research represents an enhancement in understanding the early infection mechanisms of *Anisakis* larvae in an accidental-infection model. The molecular results showed a strong localized proinflammatory response, favoring the activation of the IL17 signaling pathway and the development of the Th17-type response, with a possible co-occurrence of ribosomal stress. This is further supported by the cellular findings showing severe inflammatory/hemorrhagic lesions. Gaining an improved understanding of the effector mechanisms at the host-parasite interface lays the foundations for more focused mechanistic studies of non-adapted host-parasite interactions.

## Author contributions

IM and ŽT conceived and designed the study. Acquisition, analysis, and interpretation of data were performed by all authors (IBu, JH, AV, and IM: experimental infection and tissues sampling; IBu and JH: RNA isolation; AV: DNA isolation and RFLP; JH and IBo semi-thin sections; ŽT and IBu: RNA-Seq data analyses). The manuscript was drafted by IBu and all authors contributed to drafting sections of their area of expertise and revised it critically. All authors have read and approved the final content of the version to be published.

### Conflict of interest statement

The authors declare that the research was conducted in the absence of any commercial or financial relationships that could be construed as a potential conflict of interest.

## References

[1] Patel N Kreider T Urban JF Gause WC. Characterisation of effector mechanisms at the host:parasite interface during the immune response to tissue-dwelling intestinal nematode parasites. Int J Parasitol. (2009) 39:13–21. 10.1016/j.ijpara.2008.08.003 18804113 PMC2842902

[2] Allen JE Maizels RM. Diversity and dialogue in immunity to helminths. Nat Rev Immunol. (2011) 11:375–88. 10.1038/nri2992 21610741

[3] Anthony RM Rutitzky LI Urban JF Jr Stadecker MJ Gause WC. Protective immune mechanisms in helminth infection. Nat Rev Immunol. (2007) 7:975–87. 10.1038/nri2199 18007680 PMC2258092

[4] Zhou L Chong MMW Littman DR. Plasticity of CD4+ T cell lineage differentiation. Immunity (2009) 30:646–55. 10.1016/J.IMMUNI.2009.05.001 19464987

[5] EFSA Scientific opinion of the Panel on Biological Hazards on risk assessment of parasites in fishery products. EFSA J. (2010) 8:10–43. 10.2903/j.efsa.2010.1543.

[6] Jeon C Kim J. Pathogenic potential of two sibling species, *Anisakis simplex* (s.s.) and *Anisakis pegreffii* (Nematoda: Anisakidae): *in vitro* and *in vivo* studies. Biomed Res Int. (2015) 2015:1–9. 10.1155/2015/983656 25685821 PMC4317597

[7] Rosales MJ Mascaró C Fernandez C Luque F Moreno MS Parras L. Acute intestinal anisakiasis in Spain: a fourth-stage *Anisakis simplex* larva. Mem Inst Oswaldo Cruz (1999) 94:823–6. 10.1590/S0074-02761999000600020 10585662

[8] Baptista-Fernandes T Rodrigues M Castro I Paixão P Pinto-Marques P Roque L. Human gastric hyperinfection by *Anisakis simplex*: a severe and unusual presentation and a brief review. Int J Infect Dis. (2017) 64:38–41. 10.1016/j.ijid.2017.08.012 28882665

[9] D'amico P Malandra R Costanzo F Castigliego L Guidi A Gianfaldoni D. Evolution of the *Anisakis* risk management in the European and Italian context. Food Res Int. (2014) 64:348–62. 10.1016/j.foodres.2014.06.038 30011661

[10] Moneo I Carballeda-Sangiao N González-Muñoz M. New perspectives on the diagnosis of allergy to *Anisakis* spp. Curr Allergy Asthma Rep. (2017) 17:27. 10.1007/s11882-017-0698-x 28429304

[11] Bao M Pierce GJ Pascual S González-muñoz M. Assessing the risk of an emerging zoonosis of worldwide concern: anisakiasis. Sci Rep. (2017) 7:43699. 10.1038/srep43699 28287609 PMC5347442

[12] Bouwknegt M Devleesschauwe B Graham H Robertson LJ van der Giessen J Prioritization of foodborne parasites in Europe. Euro Surveill. (2018) 23:17–00161. 10.2807/1560-7917.ES.2018.23.9.17-00161 PMC5840924 29510783

[13] Hochberg NS Hamer DH. Anisakidosis: perils of the deep. Clin Infect Dis. (2010) 51:806–12. 10.1086/656238 20804423

[14] Ishikura H Kikuchi K Nagasawa K Ooiwa T Takamiya H Sato N. Anisakidae and anisakidosis. In: Sun T editor. Progress in Clinical Parasitology. New York, NY: Springer-Verlag (1993) p. 43–102. 10.1007/978-1-4612-2732-8_3 8420604

[15] Mattiucci S Paoletti M Colantoni A Carbone A Gaeta R Proietti A. Invasive anisakiasis by the parasite *Anisakis pegreffii* (Nematoda: Anisakidae): diagnosis by real-time PCR hydrolysis probe system and immunoblotting assay. BMC Infect Dis. (2017) 17:530. 10.1186/s12879-017-2633-0 28764637 PMC5539894

[16] Nieuwenhuizen NE. *Anisakis* – immunology of a foodborne parasitosis. Parasite Immunol. (2016) 38:548–57. 10.1111/pim.12349 27428817

[17] Audicana MT Kennedy MW. *Anisakis simplex*: from obscure infectious worm to inducer of immune hypersensitivity. Clin Microbiol Rev. (2008) 21:360–79. 10.1128/CMR.00012-07 18400801 PMC2292572

[18] Mattiucci S Paoletti M Borrini F Palumbo M Palmieri RM Gomes V. First molecular identification of the zoonotic parasite *Anisakis pegreffii* (Nematoda: Anisakidae) in a paraffin-embedded granuloma taken from a case of human intestinal anisakiasis in Italy. BMC Infect Dis. (2011) 11:82. 10.1186/1471-2334-11-82 21453522 PMC3080813

[19] Mladineo I Popović M Drmić-Hofman I Poljak V. A case report of a *Anisakis pegreffii* (Nematoda, Anisakidae) identified from archival paraffin sections of a Croatian patient. BMC Infect Dis. (2015) 16:42. 10.1186/s12879-016-1401-x 26832924 PMC4736626

[20] Zuloaga J Rodríguez-Bobada C Corcuera MT Gómez-Aguado F González P Rodríguez-Perez R. A rat model of intragastric infection with *Anisakis* spp. live larvae: Histopathological study. Parasitol Res. (2013) 112:2409–11. 10.1007/s00436-013-3359-6 23435926

[21] Gause WC Urban JF Stadecker MJ. The immune response to parasitic helminths: Insights from murine models. Trends Immunol. (2003) 24:269–77. 10.1016/S1471-4906(03)00101-7 12738422

[22] D'Amelio S Mathiopoulos KD Santos CP Pugachev ON Webb SC Picanço M. Genetic markers in ribosomal DNA for the identification of members of the genus *Anisakis* (Nematoda: Ascaridoidea) defined by polymerase-chain-reaction-based restriction fragment length polymorphism. Int J Parasitol. (2000) 30:223–6. 10.1016/S0020-7519(99)00178-2 10704605

[23] Bennett S. Solexa Ltd. Pharmacogenomics (2004) 5:433–8. 10.1517/14622416.5.4.433 15165179

[24] Bolger AM Lohse M Usadel B. Trimmomatic: a flexible trimmer for Illumina sequence data. Bioinformatics (2014) 30:2114–20. 10.1093/bioinformatics/btu170 24695404 PMC4103590

[25] Schmieder R Edwards R. Quality control and preprocessing of metagenomic datasets. Bioinformatics (2011) 27:863–4. 10.1093/bioinformatics/btr026 21278185 PMC3051327

[26] Zerbino DR Achuthan P Akanni W Amode MR Barrell D Bhai J. Ensembl 2018. Nucleic Acids Res. (2018) 46:D754–61. 10.1093/nar/gkx1098 29155950 PMC5753206

[27] Dobin A Davis CA Schlesinger F Drenkow J Zaleski C Jha S. STAR: Ultrafast universal RNA-seq aligner. Bioinformatics (2013) 29:15–21. 10.1093/bioinformatics/bts635 23104886 PMC3530905

[28] Robinson MD McCarthy DJ Smyth GK. edgeR: a Bioconductor package for differential expression analysis of digital gene expression data. Bioinformatics (2010) 26:139–40. 10.1093/bioinformatics/btp616 19910308 PMC2796818

[29] McCarthy DJ Chen Y Smyth GK. Differential expression analysis of multifactor RNA-Seq experiments with respect to biological variation. Nucleic Acids Res. (2012) 40:4288–97. 10.1093/nar/gks042 22287627 PMC3378882

[30] R Development Core Team R: A Language and Environment for Statistical Computing (2017). Available online at: https://www.r-project.org/

[31] Huber W Carey VJ Gentleman R Anders S Carlson M Carvalho BS. Orchestrating high-throughput genomic analysis with Bioconductor. Nat Methods (2015) 12:115–21. 10.1038/nmeth.3252 25633503 PMC4509590

[32] Carlson M Org.Rn.eg.db: Genome Wide Annotation for Rat. R Package Version 3.4.2 (2017).

[33] McCarthy DJ Smyth GK. Testing significance relative to a fold-change threshold is a TREAT. Bioinformatics (2009) 25:765–71. 10.1093/bioinformatics/btp053 19176553 PMC2654802

[34] Smyth GK. Linear models and empirical Bayes methods for assessing differential expression in microarray experiments. Stat Appl Genet Mol Biol. (2004) 3:1–25. 10.2202/1544-6115.1027 16646809

[35] Gu Z Eils R Schlesner M. Complex heatmaps reveal patterns and correlations in multidimensional genomic data. Bioinformatics (2016) 32:2847–9. 10.1093/bioinformatics/btw313 27207943

[36] Walter W Sánchez-Cabo F Ricote M. GOplot: An R package for visually combining expression data with functional analysis. Bioinformatics (2015) 31:2912–4. 10.1093/bioinformatics/btv300 25964631

[37] Wickham H Ggplot2 - Elegant Graphics for Data Analysis. Use R!. New York, NY: Springer (2009).

[38] Kanehisa M Furumichi M Tanabe M Sato Y Morishima K. KEGG: new perspectives on genomes, pathways, diseases and drugs. Nucleic Acids Res. (2017) 45:D353–61. 10.1093/nar/gkw1092 27899662 PMC5210567

[39] Luo W Friedman MS Shedden K Hankenson KD Woolf PJ. GAGE: generally applicable gene set enrichment for pathway analysis. BMC Bioinformatics (2009) 10:161. 10.1186/1471-2105-10-161 19473525 PMC2696452

[40] Luo W Brouwer C. Pathview: an R/Bioconductor package for pathway-based data integration and visualization. Bioinformatics (2013) 29:1830–1. 10.1093/bioinformatics/btt285 23740750 PMC3702256

[41] Fumarola L Monno R Ierardi E Rizzo G Giannelli G Lalle M *Anisakis pegreffii* etiological agent of gastric infections in two Italian women. Foodborne Pathog Dis. (2009) 6:1157–9. 10.1089/fpd.2009.0325 19642920

[42] Mattiucci S Fazii P Rosa A De Paoletti M Megna AS Glielmo A. Anisakiasis and gastroallergic reactions associated with *Anisakis pegreffii* infection, Italy. Emerg Infect Dis. (2013) 19:496–9. 10.3201/eid1903.121017 23621984 PMC3647659

[43] Baird FJ Su X Aibinu I Nolan MJ Sugiyama H Otranto D. The *Anisakis* transcriptome provides a resource for fundamental and applied studies on allergy-causing parasites. PLoS Negl Trop Dis. (2016) 10:e0004845. 10.1371/journal.pntd.0004845 27472517 PMC4966942

[44] Cavallero S Lombardo F Su X Salvemini M Cantacessi C D'Amelio S. Tissue-specific transcriptomes of *Anisakis simplex* (*sensu stricto*) and *Anisakis pegreffii* reveal potential molecular mechanisms involved in pathogenicity. Parasit Vectors (2018) 11:31. 10.1186/s13071-017-2585-7 29321072 PMC5763927

[45] Baeza ML Conejero L Higaki Y Martín E Pérez C Infante S. *Anisakis simplex* allergy: a murine model of anaphylaxis induced by parasitic proteins displays a mixed Th1/Th2 pattern. Clin Exp Immunol. (2005) 142:433–40. 10.1111/j.1365-2249.2005.02952.x 16297154 PMC1809526

[46] Gonzalez-Muñoz M Rodriguez-Mahillo AI Moneo I. Different Th1/Th2 responses to *Anisakis simplex* are related to distinct clinical manifestations in sensitized patients. Parasite Immunol. (2010) 32:67–73. 10.1111/j.1365-3024.2009.01162.x 20042009

[47] Park H Li Z Yang XO Chang SH Nurieva R Wang Y-H. A distinct lineage of CD4 T cells regulates tissue inflammation by producing interleukin 17. Nat Immunol. (2005) 6:1133–41. 10.1038/ni1261 16200068 PMC1618871

[48] Weaver CT Hatton RD Mangan PR Harrington LE. IL-17 family cytokines and the expanding diversity of effector T cell lineages. Annu Rev Immunol. (2007) 25:821–52. 10.1146/annurev.immunol.25.022106.141557 17201677

[49] Proskuryakov SY Konoplyannikov AG Gabai VL. Necrosis: A specific form of programmed cell death? Exp Cell Res. (2003) 283:1–16. 10.1016/S0014-4827(02)00027-7 12565815

[50] Junqueira LC Carneiro J Basic Histology: Text & Atlas. 11th edn. New York, NY: McGraw-Hill, Medical Pub. Division (2005).

[51] Cho MK Park MK Kang SA Caballero ML Perez-Pinar T Rodriguez-Perez R. Allergenicity of two *Anisakis simplex* allergens evaluated *in vivo* using an experimental mouse model. Exp Parasitol. (2014) 146:71–7. 10.1016/j.exppara.2014.09.008 25300761

[52] Abdel-Ghaffar F Badr AM Morsy K Ebead S El Deeb S Al Quraishy S. Cytokine signature and antibody-mediated response against fresh and attenuated *Anisakis simplex* (L3) administration into Wistar rats: implication for anti-allergic reaction. Parasitol Res. (2015) 114:2975–84. 10.1007/s00436-015-4500-5 25982570

[53] Messina CM Pizzo F Santulli A Bušelić I Boban M Orhanović S. *Anisakis pegreffii* (Nematoda: Anisakidae) products modulate oxidative stress and apoptosis-related biomarkers in human cell lines. Parasit Vectors (2016) 9:607. 10.1186/s13071-016-1895-5 27887635 PMC5124272

[54] Napoletano C Mattiucci S Colantoni A Battisti F Zizzari IG Rahimi H. *Anisakis pegreffii* impacts differentiation and function of human dendritic cells. Parasite Immunol. (2018) 40:e12527. 10.1111/pim.12527 29569735

[55] Murphy K Weaver C Janeway's Immunobiology. New York, NY: Garland Science, Taylor & Francis Group, LLC (2017)

[56] Kang SA Cho MK Park M-K Kim D-H Hong YC Lee YS. Alteration of helper T-cell related cytokine production in splenocytes during *Trichinella spiralis* infection. Vet Parasitol. (2012) 186:319–27. 10.1016/J.VETPAR.2011.12.002 22222009

[57] Katz M Despommier DD Gwadz R Parasitic Diseases. New York, NY: Springer-Verlag (1989).

[58] Murphy CA Langrish CL Chen Y Blumenschein W McClanahan T Kastelein RA. Divergent pro- and antiinflammatory roles for IL-23 and IL-12 in joint autoimmune inflammation. J Exp Med. (2003) 198:1951–7. 10.1084/jem.20030896 14662908 PMC2194162

[59] Yen D Cheung J Scheerens H Poulet F McClanahan T Mckenzie B. IL-23 is essential for T cell-mediated colitis and promotes inflammation via IL-17 and IL-6. J Clin Invest. (2006) 116:1310–6. 10.1172/JCI21404 16670770 PMC1451201

[60] Zheng Y Danilenko DM Valdez P Kasman I Eastham-Anderson J Wu J Interleukin-22, a Th17 cytokine, mediates IL-23-induced dermal inflammation and acanthosis. Nature (2007) 445:648–51. 10.1038/nature05505 17187052

[61] Johansson MEV Phillipson M Petersson J Velcich A Holm L Hansson GC. The inner of the two Muc2 mucin-dependent mucus layers in colon is devoid of bacteria. PNAS (2008) 105:15064–9. 10.1073/pnas.0803124105 18806221 PMC2567493

[62] Swidsinski A Weber J Loening-baucke V Hale LP Lochs H. Spatial organization and composition of the mucosal flora in patients with inflammatory bowel disease. J Clin Microbiol. (2005) 43:3380–9. 10.1128/JCM.43.7.3380 16000463 PMC1169142

[63] Vaishnava S Yamamoto M Severson KM Ruhn KA Yu X Koren O. The antibacterial lectin RegIIIγ promotes the spatial segregation of microbiota and host in the intestine. Science (2011) 334:255–8. 10.1126/science.1209791 21998396 PMC3321924

[64] Jin W Dong C. IL-17 cytokines in immunity and inflammation. Emerg Microbes Infect. (2013) 2:e60. 10.1038/emi.2013.58 26038490 PMC3820987

[65] Pathak M Verma M Srivastava M Misra-Bhattacharya S. *Wolbachia* endosymbiont of *Brugia malayi* elicits a T helper type 17-mediated pro-inflammatory immune response through *Wolbachia* surface protein. Immunology (2015) 144:231–44. 10.1111/imm.12364 25059495 PMC4298417

[66] Anuradha R Munisankar S Dolla C Kumaran P Nutman TB Babu S. Parasite antigen-specific regulation of Th1, Th2, and Th17 responses in *Strongyloides stercoralis* infection. J Immunol. (2015) 195:2241–50. 10.4049/jimmunol.1500745 26202988 PMC4546867

[67] Bateman A Martin MJ O'Donovan C Magrane M Alpi E Antunes R UniProt: the universal protein knowledgebase. Nucleic Acids Res. (2017) 45:D158–69. 10.1093/nar/gkw1099 27899622 PMC5210571

[68] Karadjian G Fercoq F Pionnier N Vallarino-Lhermitte N Lefoulon E. Migratory phase of *Litomosoides sigmodontis* filarial infective larvae is associated with pathology and transient increase of S100A9 expressing neutrophils in the lung. PLoS Negl Trop Dis. (2017) 11:e0005596. 10.1371/journal.pntd.0005596 28486498 PMC5438187

[69] Page-McCaw A. Remodeling the model organism: matrix metalloproteinase functions in invertebrates. Semin Cell Dev Biol. (2008) 19:14–23. 10.1016/j.semcdb.2007.06.004 17702617 PMC2248213

[70] Parks WC Parks WC Wilson CL Wilson CL López-Boado YS López-Boado YS. Matrix metalloproteinases as modulators of inflammation and innate immunity. Nat Rev Immunol. (2004) 4:617–29. 10.1038/nri1418 15286728

[71] Angele MK Knöferl MW Ayala A Albina JE Cioffi WG Bland KI. Trauma-hemorrhage delays wound healing potentially by increasing pro-inflammatory cytokines at the wound site. Surgery (1999) 126:279–85. 10.1016/S0039-6060(99)70166-2 10455895

[72] O'Connell EM Nutman TB. Eosinophilia in infectious diseases. Immunol Allergy Clin North Am. (2015) 35:493–522. 10.1016/j.iac.2015.05.003 26209897 PMC4515572

[73] Martin P Leibovich SJ. Inflammatory cells during wound repair: The good, the bad and the ugly. Trends Cell Biol. (2005) 15:599–607. 10.1016/j.tcb.2005.09.002 16202600

[74] Shirey KA Pletneva LM Puche AC Keegan AD Gregory A Blanco JCG Control of RSV-induced lung injury by alternatively activated macrophages is IL-4Rα-, TLR4-, and IFN-β-dependent. Mucosal Immunol. (2010) 3:291–300. 10.1038/mi.2010.6.Control 20404812 PMC2875872

[75] Nembrini C Marsland BJ Kopf M. IL-17-producing T cells in lung immunity and inflammation. J Allergy Clin Immunol. (2009) 123:986–94. 10.1016/j.jaci.2009.03.033 19410688

[76] Cooper AM. IL-17 and anti-bacterial immunity: protection *versus* tissue damage. Eur J Immunol. (2009) 39:649–52. 10.1002/eji.200839090 19283706 PMC3931122

[77] Naka T Nishimoto N Kishimoto T. The paradigm of IL-6: from basic science to medicine. Arthritis Res. (2002) 4:S233. 10.1186/ar565 12110143 PMC3240141

[78] Mangan PR Harrington LE O'Quinn DB Helms WS Bullard DC Elson CO Transforming growth factor-β induces development of the Th17 lineage. Nature (2006) 441:231–4. 10.1038/nature04754 16648837

[79] Veldhoen M Hocking RJ Atkins CJ Locksley RM Stockinger B. TGFβ in the context of an inflammatory cytokine milieu supports de novo differentiation of IL-17-producing T cells. Immunity (2006) 24:179–89. 10.1016/J.IMMUNI.2006.01.001 16473830

[80] Cho M-L Kang J-W Moon Y-M Nam H-J Jhun J-Y Heo S-B. STAT3 and NF-κB signal pathway is required for IL-23-mediated IL-17 production in spontaneous arthritis animal model IL-1 receptor antagonist-deficient mice. J Immunol. (2006) 176:5652–61. 10.4049/jimmunol.176.9.5652 16622035

[81] Pedersen BK Febbraio MA. Muscle as an endocrine organ. Physiol Rev. (2008) 88:1379–406. 10.1152/physrev.90100.2007 18923185

[82] Muñoz-Cánoves P Scheele C Pedersen BK Serrano AL. Interleukin-6 myokine signaling in skeletal muscle: a double-edged sword? FEBS J. (2013) 280:4131–48. 10.1111/febs.12338 23663276 PMC4163639

[83] Zhou X Liao WJ Liao JM Liao P Lu H. Ribosomal proteins: Functions beyond the ribosome. J Mol Cell Biol. (2015) 7:92–104. 10.1093/jmcb/mjv014 25735597 PMC4481666

[84] Mirhashemi ME Noubary F Chapman-Bonofiglio S Tzipori S Huggins GS Widmer G. Transcriptome analysis of pig intestinal cell monolayers infected with *Cryptosporidium parvum* asexual stages. Parasit Vectors (2018) 11:176. 10.1186/s13071-018-2754-3 29530089 PMC5848449

