# Supplementary material for: Molecular and Cellular Response to Experimental Anisakis pegreffii (Nematoda, Anisakidae) Third-Stage Larval Infection in Rats
Source: Front Immunol. 2018 Sep 7;9:2055. doi: 10.3389/fimmu.2018.02055 (PMC6137129; doi:10.3389/fimmu.2018.02055)
Supplement: Supplementary file 5 [file Data_Sheet_1.pdf]

Supplementary Material

Molecular and Cellular Response to Experimental *Anisakis pegreffii* (Nematoda, Anisakidae) Third-Stage Larval Infection in Rats

Ivana Bušelić[1*†], Željka Trumbić[2†], Jerko Hrabar[1], Anamarija Vrbatović[1], Ivana Bočina[3], Ivona Mladineo[1]

[1] Institute of Oceanography and Fisheries, Split, Croatia, [2] Department of Marine Studies, University of Split, Split, Croatia, [3] Faculty of Science, University of Split, Split, Croatia

[†]Authors contributed equally to this manuscript.

[*]Correspondence:

Ivana Bušelić

buselic@izor.hr

Supplementary Material

Supplementary Table 1. Detailed description of pooled samples for RNA-Seq, showing the state of infection, the number of biological replicates in the pool, pairing with complementary control tissues, sampling hour and description of the biological replicates. Samples originating from rats intubated with saline are marked as Uninfected.

Supplementary Table 2. Complete list of differentially expressed genes (FDR < 0.05) in *Anisakis*-infected stomach and muscle of rats (separate Excel sheets), with Entrez Gene ID, gene description, gene symbol, log2 fold change value (logFC), log2-counts-per-million value (logCPM), p value, FDR and TREAT (marked with True if the gene survived the TREAT test and with False if not). All the genes that survived the TREAT test are additionally marked in bold.

Supplementary Table 3. Overrepresented gene ontology (GO) terms for *Anisakis*-infected stomach and muscle of rats (separate Excel sheets) using goana function built-in edgeR. For each GO term is given sub-ontology (biological process (BP), cellular component (CC), and molecular function (MF)), number of genes included in the GO term, number of up-regulated genes in our dataset included in the GO term, p value and FDR.

Supplementary Table 4. Significantly perturbed KEGG signaling, metabolic and disease-related pathways in *Anisakis*-infected stomach and muscle of rats (separate Excel sheets) using GAGE analyses (q < 0.1). Non-redundant pathways (do not show high overlap in their effective gene list) are

shown in bold. For each pathway is given type, dataset, direction, pathway ID, statistical mean, p value, q value and set size.

Supplementary Figure 1. Visualization of the first two components of principal component analyses performed on log2-counts-per-million (logCPM) normalized values of host tissues (stomach and muscle) affected by the larval migration (*Anisakis*), adjoining unaffected tissues of experimentally infected Sprague-Dawley rats (Control) and the same tissues of uninfected rats (Uninfected).

Supplementary Figure 2. Visualization of MA plots showing differentially expressed genes in red and differentially expressed genes passing the TREAT test (|logFC| >> 1) are marked with gene symbols.
