# Supplementary figures and images for: Molecular and Cellular Response to Experimental Anisakis pegreffii (Nematoda, Anisakidae) Third-Stage Larval Infection in Rats
Source: Front Immunol. 2018 Sep 7;9:2055. doi: 10.3389/fimmu.2018.02055 (PMC6137129; doi:10.3389/fimmu.2018.02055)

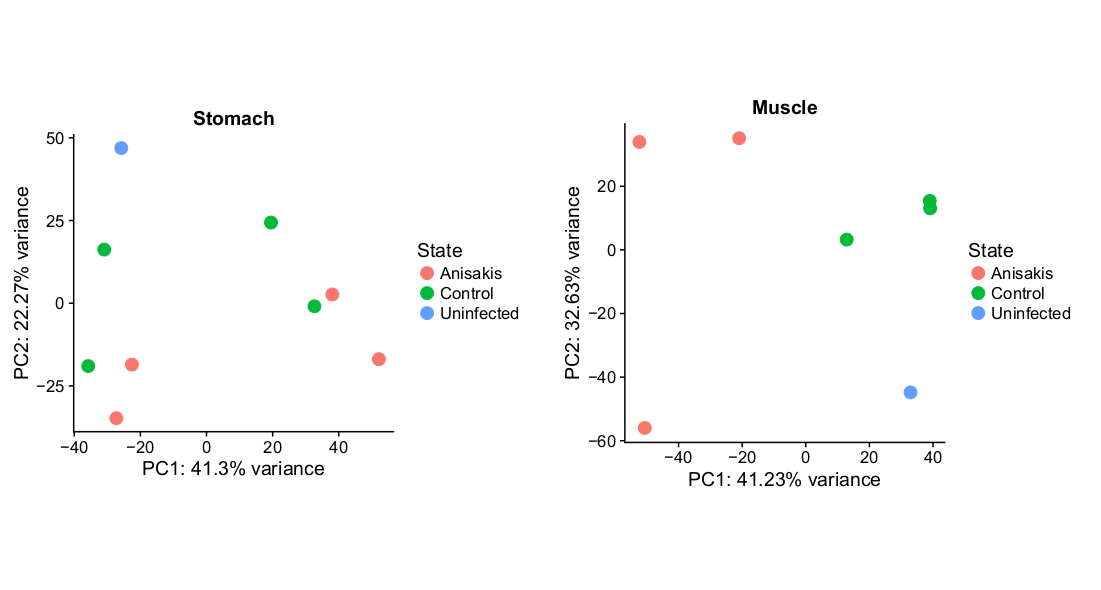

Supplement: Supplementary file 6 [file Image_1.tif]

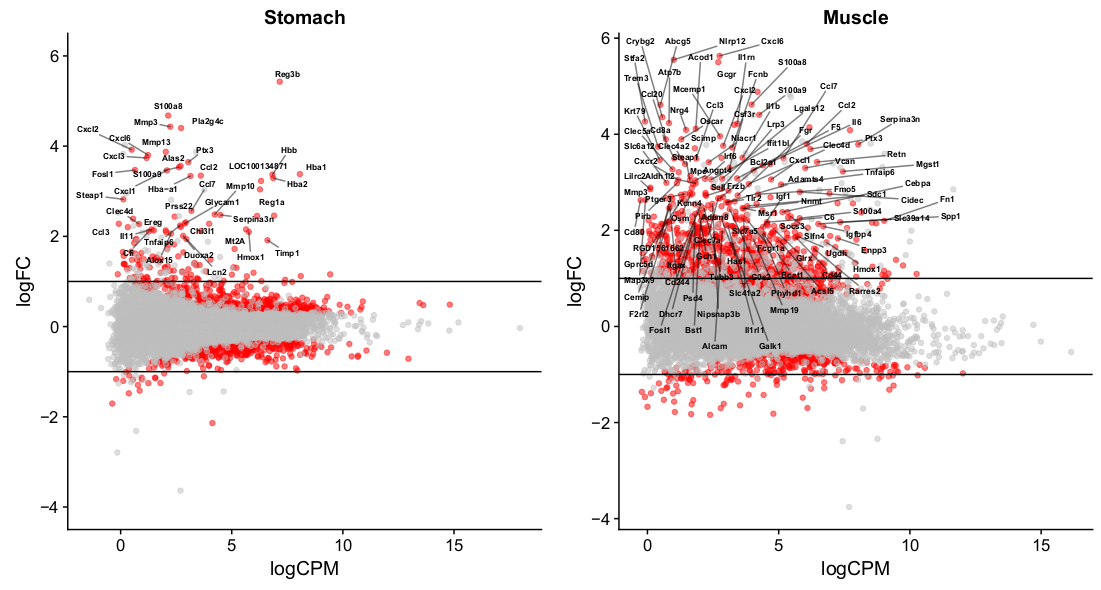

Supplement: Supplementary file 7 [file Image_2.tif]
